# Initial Experience With a Bioresorbable Polymer Anchor

**DOI:** 10.7759/cureus.12370

**Published:** 2020-12-29

**Authors:** Robert R Burnham, Jayanth Kumar, Michael Pinzur, Adam Schiff

**Affiliations:** 1 Orthopaedic Surgery, Loyola University Medical Center, Maywood, USA; 2 Orthopaedic Surgery, Loyola University Chicago Stritch School of Medicine, Maywood, USA

**Keywords:** soft tissue fixation, lateral ankle ligament instability, deltoid ligament repair, bioresorbable implant, polymer implant, sonicanchor

## Abstract

Background

Anchors are frequently used in reconstructive orthopedic surgery to achieve fixation of soft tissue to bone. Anchors vary with respect to material composition, configuration, and methods of fixation at the site of attachment. The fixation component of anchoring devices has generally evolved from metal-fabricated implants to various types of bioresorbable anchors. The SonicAnchor^TM^ (Stryker, Kalamazoo, MI USA) polymer implant provides a unique form of anchor fixation using SonicFusion^TM^ technology to achieve interdigitation within cancellous bone while being radiolucent and providing a small footprint.

Methods

During a four-year period, 116 patients underwent a reconstructive orthopedic foot and ankle surgical procedure with the use of at least one bioresorbable polymer anchor (SonicAnchor implant). There were 59 males and 57 females, with an average age of 42 years (range: 12-83 years).

Results

A total of 233 bioresorbable anchor (SonicAnchor) implants were used in 116 patients. Of the 116 patients, 108 (93.1%) achieved successful clinical healing of their surgery at their most recent follow-up. The average follow-up duration was 309 days (range: 14-1,429 days). Eight (6.9%) patients were lost to follow-up prior to clinical healing. Two (1.7%) patients underwent reoperation. Also, 65 (56%) patients had at least six months of follow-up and 36 (31%) had at least one year of follow-up.

Conclusions

This preliminary clinical trial of patients undergoing soft tissue repair or reconstruction with a bioresorbable polymer appears to perform comparably to other commercially available devices. The lack of adverse events, mechanical failures, or infections further supports the safety of this device.

## Introduction

The quality of fixation of tendon or ligament to bone is a crucial consideration when selecting surgical anchors to perform soft tissue repair or reconstruction for musculoskeletal injuries [1]. Anchors have supplanted drill holes as an accepted method of surgically securing soft tissue to bone temporarily until definitive healing to bone occurs. Historically, initial anchors were composed of metal, but more recently they have evolved to radiolucent biopolymers [2-7]. The suture anchor has become an increasingly utilized method for suspensory fixation due to its ability to provide stronger fixation than punch-in anchors, as well as comparable strength and displacement to a knotted anchor [8-10].

Traditional anchors were made of metal. Imaging concerns coupled with technical complications prompted a shift to polymer-based implants [11-14]. While generally well accepted, polymer anchors occasionally fail due to material failure or loss of fixation caused by premature degradation [15-17]. A bioresorbable polymer composed of poly(L-lactide-co-D,L-lactide) was used to fabricate a biopolymer anchor with potential for enhanced fixation to cancellous bone (SonicAnchor^TM^, Stryker, Kalamazoo, MI, USA). This soft tissue anchor utilizes controlled ultrasonic energy (SonicFusion^TM^, Stryker) technology to “liquefy” the polymer anchor after an initial unicortical pilot hole has been drilled. The anchor then quickly reconstitutes within the cancellous bone allowing for enhanced interdigitation of the implant [18]. Recent biomechanical investigations have demonstrated enhanced strength of fixation to bone using this technology compared to popular, commercially available implants [19-21]. The goal of this preliminary trial was to collect and analyze clinical follow-up data in a consecutive series of patients undergoing repair or reconstruction for soft tissue injuries in the foot and ankle using this biopolymer anchor.

## Materials and methods

Following Institutional Review Board approval, a retrospective chart review was performed on 116 consecutive patients undergoing soft tissue repair or reconstruction for injuries or disorders in the foot and ankle. A biopolymer, resorbable anchor (SonicAnchor) was used to secure fixation of soft tissue to bone. All patients who received at least one of these biopolymer, resorbable anchors were included in this study. There were no specific exclusion criteria for this study. Surgery was performed by two orthopedic foot and ankle surgeons in an academic medical center during a 3.5-year period from December 2015 to June 2019. Charts were abstracted for demographics and comorbidities, and surgery was performed. The success of surgery was defined as return to activities as tolerated with no restrictions or assistive devices. Adverse events included perioperative complications, infections, and failure to achieve the goals of the surgery.

## Results

A total of 233 biopolymer resorbable implants (SonicAnchor) were implanted in 116 consecutive patients. There were 57 female and 59 male patients, with an average age of 42 years (range: 12-83 years). The average BMI was 30.76 (standard deviation: 8.17) kg/m^2^. Six patients were diabetic, 13 were current smokers, and 20 were previous smokers.

Table 1 demonstrates the primary diagnoses of patients who received at least one SonicAnchor bioresorbable suture anchor. Lateral ligamentous instability (58) and medial/deltoid ligamentous instability (49) were among the most common diagnoses. The operative procedures performed are listed in Table 2. The most common procedures were lateral ankle ligament reconstruction (38), deltoid ligament repair (33), and tendon repair (27).

**Table 1:** Primary Diagnoses

Primary Diagnoses	
Lateral ligamentous instability	58
Medial (deltoid) ligamentous instability	49
Chronic ankle instability	28
Achilles injury or equinus contracture	16
Accessory navicular	8
Foot drop	2
Syndesmotic injury	16
Haglund's deformity	13

**Table 2:** Operative Procedures

Operative Procedures	
Tendon repair	27
Tendon advancement	3
Achilles repair/lengthening	15
Lateral ankle ligament reconstruction	38
Tendon transfer	21
Deltoid ligament repair	33

Of the 116 patients, 108 (93.1%) achieved clinical healing. Eight (6.9%) patients were lost to follow-up prior to demonstrating clinical healing. All 108 patients who had adequate follow-up achieved clinical healing. Sixty-five (56%) patients had at least six months of follow-up and 36 (31%) had at least one year of follow-up (Table 3). Average follow-up duration was 309 days (range: 14-1,429 days). Two patients who followed up at their two-week visit for suture removal were lost to follow-up. The next shortest follow-up duration was at 41 days.

**Table 3:** Patient Follow-Up

Follow-Up	
Patients with clinical healing	108 (93.1%)
Lost to follow-up prior to clinical healing	8 (6.9%)
Reoperation	2 (1.7%)
Patients with six months of follow-up	65 (56%)
Patients with one year of follow-up	36 (31%)
Total patients	116

Three patients required reoperation, with only one requiring revision secondary to persistent chronic instability. This patient was a 48-year-old female with a history of ankle dislocation and subsequent development of chronic lateral ligament instability. She achieved clinical success following a second surgery consisting of lateral ankle ligament reconstruction with an allograft. Another reoperation patient was taken back to surgery as he sustained a bi-malleolar ankle fracture after falling from a tree. The patient demonstrated clinical healing by returning to full activities without restrictions and no pain at rest or with activity. This injury occurred eight months after his initial lateral ankle ligament reconstruction. The third patient had a history of foot drop for which she underwent a tibialis posterior tendon transfer. Her tendon transfer had stretched out over the course of 1.5 years and was prohibiting her from achieving neutral ankle dorsiflexion. She underwent an Achilles tendon lengthening and peroneus brevis and longus transfers to the dorsum of her midfoot.

Of the three reoperations, persistent chronic instability in one patient could be attributed to the failure of the biopolymer implant, but more likely due to the surgical technique. We believe the patient who sustained a bi-malleolar ankle fracture was a separate event that occurred after the biopolymer implant had already achieved clinical healing. The patient with a foot drop who underwent a tibialis posterior tendon transfer may have also been a result of implant failure. Although the tendon may have failed anywhere along its length, we could not exclude the possibility that the tendon may have failed to incorporate at the site of the implant. Of all the reoperations, none of the biopolymer implants was removed.

## Discussion

**Figure 1:**
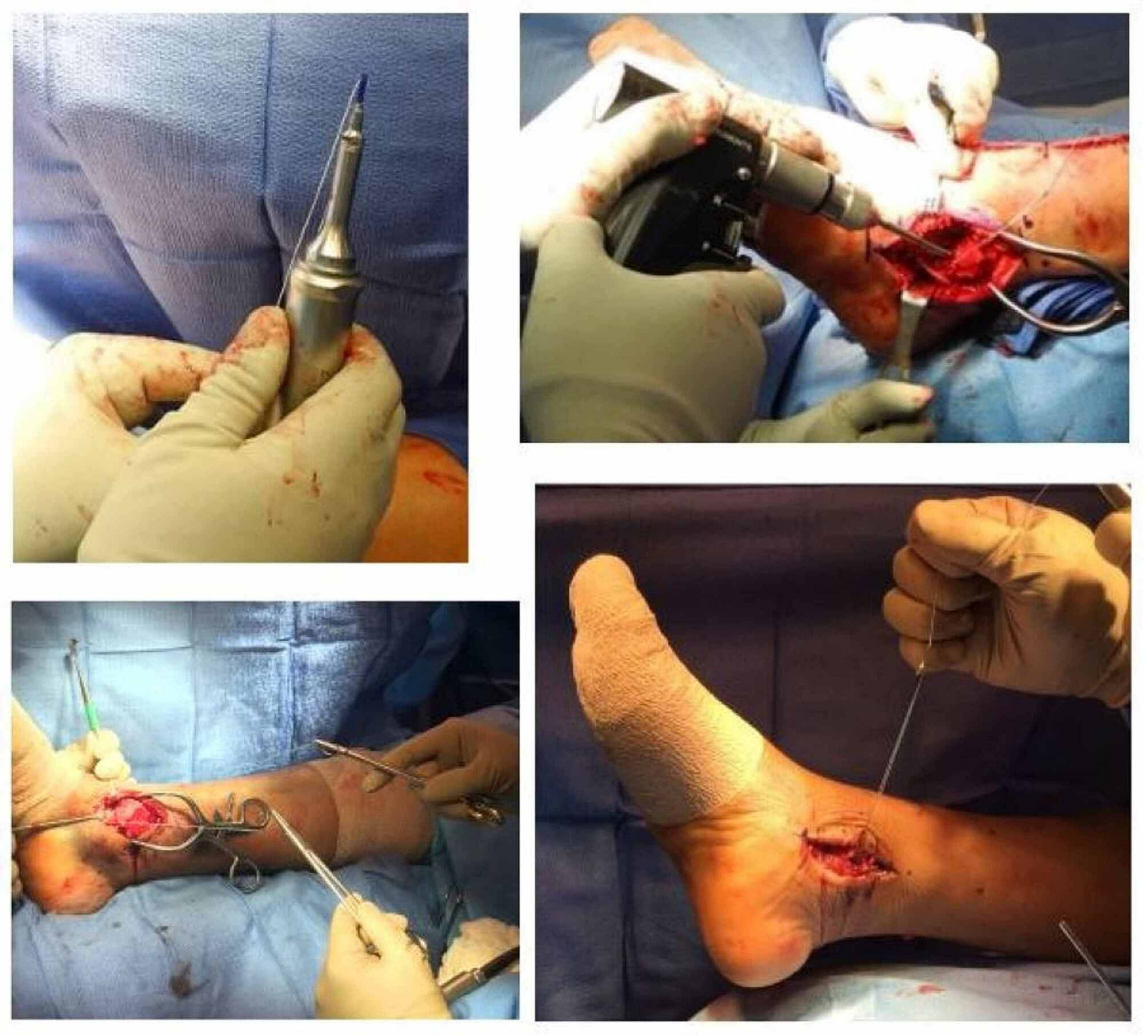
Clinical images of the bioresorbable polymer suture anchor. Top left: The insertion apparatus with a suture anchor attached at the tip of the device. Top right: Drilling of the desired suture anchor location. Bottom left: The suture anchor has been liquefied and has interdigitated within the cancellous bone. Two suture needles are held by needle drivers to be sewn into the desired tissue. Bottom right: The surgeon is picking up the weight of the lower extremity using the newly placed suture anchors, which demonstrates the strength of this suture anchor fixation.

Suture anchors have evolved as the preferred method of securing ligament or tendon to bone in reconstructive musculoskeletal surgery. Surgical anchoring has evolved from drill holes to metal suture anchors to the current preference for radiolucent biopolymer suture anchors. The bioresorbable polymer device used in this preliminary investigation (SonicAnchor) was created using a technology that can enhance the structural stability of the soft tissue anchor [21,22]. The goal was to create an anchor that could be “liquefied” and quickly reconstituted to achieve enhanced pullout strength and resistance to fatigue failure [19,23]. Figure 1 shows the short and simple steps for placing this bioresorbable anchor. This retrospective investigation suggests that the device appears to be safe and effective for use in orthopedic foot and ankle procedures in the short- and mid-term time frame.

One major drawback of using these novel bioresorbable polymer anchors is cost. The cost of each individual anchor varies based on the costs negotiated by individual hospitals. In contrast, the use of transosseous fixation in the form of bone tunnels requires no additional tools or implants. However, further studies need to be conducted to compare the rate of reoperation, clinical success, operative time, and cost of each method of fixation to truly evaluate the cost-effectiveness of each method.

The results of this retrospective case series compare favorably with similar ultrasonically interdigitated anchor use in a similar cohort of patients when examining the safety and efficacy profile [24,25].

The primary limitation to this preliminary clinical trial is the retrospective nature of this study. In addition, eight patients were lost to follow-up prior to demonstrating clinical healing. Two of the eight patients were lost to follow-up after their two-week postoperative visit. All patients were graduated from follow-up after they had demonstrated evidence of clinical healing, which limited the ability to evaluate patient status at long-term follow-up intervals. Follow-up for this study was highly influenced by surgeon preference to allow patients to follow-up on an as-needed basis after they had demonstrated clinical healing at their follow-up. Ideal future studies should include long-term follow-up and functional outcome scores.

## Conclusions

This preliminary clinical trial of consecutive patients undergoing soft tissue repair or reconstruction with a novel bioresorbable suture anchor appeared to demonstrate a favorable safety and clinical effectiveness profile. The low incidence of adverse events (two of 116, 1.7%), mechanical failures, or infections support the claims of this device. Further clinical studies need to be performed to support the perceived benefits of this radiolucent bioresorbable polymer suture anchor compared to other commercially available anchors.
